# Bycatch and discards in the artisanal shrimp trawl fishery in Northern Peru

**DOI:** 10.1371/journal.pone.0268128

**Published:** 2022-06-22

**Authors:** Jaime Mendo, Tania Mendo, Patricia Gil-Kodaka, Jimmy Martina, Iván Gómez, Ruggeri Delgado, Jhenifer Fernández, Alejandra Travezaño, Ruggeri Arroyo, Karla Loza, Mark A. James

**Affiliations:** 1 Facultad de Pesquería, Universidad Nacional Agraria La Molina, Lima, Peru; 2 Scottish Oceans Institute, University of St Andrews, Fife, United Kingdom; COISPA Tecnologia & Ricerca - Stazione Sperimentale per lo Studio delle Risorse del Mare, ITALY

## Abstract

This study analyses the bycatch composition of an artisanal shrimp trawl fleet operating between Cabo Blanco and Máncora in Northern Peru between April 2019 and March 2020. A total of 300 hauls were analysed with respect to target catch and bycatch (consisting of other commercial species, discards, and macroalgae). A total of 277 species were recorded including 111 species of fish, 65 species of molluscs, 51 species of crustaceans, 22 species of algae, 12 species of cnidarian, 9 species of echinoderms, 4 species of Bryozoa and 3 species of polychaeta. Capture per unit effort (CPUE, kg.h^-1^) was highest for fish, followed by crustaceans, algae and molluscs. The target species *Penaeus californiensis* coffee shrimp constituted 17.8% of the overall catch,82.2% represented bycatch, and 50.6% represented discards. Coffee shrimps were more abundant in June and November 2019 and in January and February 2020. Highest bycatch CPUE occurred in May, June and December 2019. The most abundant species in the bycatch throughout the study period were sand perch *(Diplectrum conceptione*, *16% weight of the total catch)*, the macroalgae caulerpa (*Caulerpa filiformis*, *13%)*, sole flounder *(Etropus ectenes*, *6*.*4%)*, Pacific drum *(Larimus pacificus*, *5*.*7%*), and lumptail searobin (*Prionotus stephanophrys*, *5*.*1%*). Overall, the contribution of sand perch and flounder, exceeded the weight of coffee shrimp, therefore the interpretation that shrimp is the sole target species needs to be revised. The number of discarded species per month increased towards the spring months with the highest value in November. This study represents the first characterisation of bycatch in the artisanal trawling fishery in the Piura region in northern Peru and reveals a high proportion of bycatch in the fishery but also hints at potential temporal management measures that could be imposed to reduce the levels of bycatch. For example, the months of May and December had the greatest bycatch to shrimp ratios and the fishery could potentially be closed to avoid high bycatch risk, however, longer term information is needed to assess if the trends observed in bycatch are similar over longer periods of time. The species characterisation of bycatch also provides information for the design of modified nets which would target the reduction of small fish present in the catch.

## Introduction

Bottom trawling and dredging inflict one of the most damaging impacts on seabed habitats [1–4]. Bottom trawling often results in the capture of juveniles of many commercial and non-commercial species which are discarded. It is estimated that bottom trawl fisheries contribute to 45% of all discards 4.2 million tonnes [5]. The impacts of this practice depend on the complexity and natural variability of these communities and the nature of the fishery [6, 7].

In Peru, only small-scale fisheries using gears not considered to have a high impact on the ecosystem are allowed to operate within 5 miles of the coast (General Fisheries Law 2001, DS-012-2001-PE). Despite this regulation, small scale shrimp trawlers operate mainly within coastal areas. In the 1960s this fishery consisted of 12 small vessels with 10–15 m^3^ hold capacity. These vessels mainly fished for shrimp in northern Peru, between Paita (Piura region,) and Puerto Pizarro (Tumbes region) [8]. According to the I National Census of Artisanal Fisheries, by 2012, there were 88 vessels operating in the Tumbes Region and 55 in the Piura Region [9]. Currently it is estimated that around 105 vessels operate in the Piura region [10]. Whilst these vessels are fishing illegally, policing is sporadic and ineffectual. Evidence suggests that the fishery makes a significant socio-economic contribution to the local economy [10], albeit with impacts on biodiversity that characterises this type of fishing. The illegal nature of this fishery has so far prevented management or monitoring of this fishery by the administration and therefore there is no detailed information on the composition of bycatch, or the magnitude of discards that, according to [11], constitute 95% of the total catch in the Tumbes region.

There is limited information about the level of impact of the shrimp trawl fishery in Piura. A preliminary report by [12] provides a 15-day survey on shrimp landings and commercial bycatch for one fishing vessel operating in this region and showed a bycatch rate of 93.3% of the total catch weight. Fishers in Piura have repeatedly requested that the Peruvian Marine Institute (IMARPE) conduct research to reduce the level of impact of this fishery (pers. com. Alex Eche, president of fisher’s organisation) and find sustainable measures that would allow the fishery to be formally regulated. In this context, IMARPE conducted an experimental fishery in Sechura Bay and Talara (Piura) to assess the selectivity and impact of the shrimp trawl fishery on biodiversity and the seabed [13]. The objective of this experimental fishery was to design a net that would reduce the levels of bycatch. However, only 16 hauls were recorded during one month of sampling and no new design was provided to fishers. In this context, fishers contacted members of the National Agrarian University, to again pursue ways to increase the sustainability of the fishery. In this context, the DYNAMICOPERU project has contributed to the biological, fisheries, technological and economic knowledge that would allow, through a participatory approach, the identification of management strategies to reduce the impact of this fishery on biodiversity. This study analyses the composition of the total catch, bycatch and discards of this fisheries operating between Cabo Blanco and Máncora (Piura region) from April 2019 –March 2020, as a basis for assessing the impact on biodiversity and sustainability of populations within the 5-mile trawling exclusion area. Strategies to reduce the impact of shrimp trawling are discussed.

## Methods

**Fig 1:**
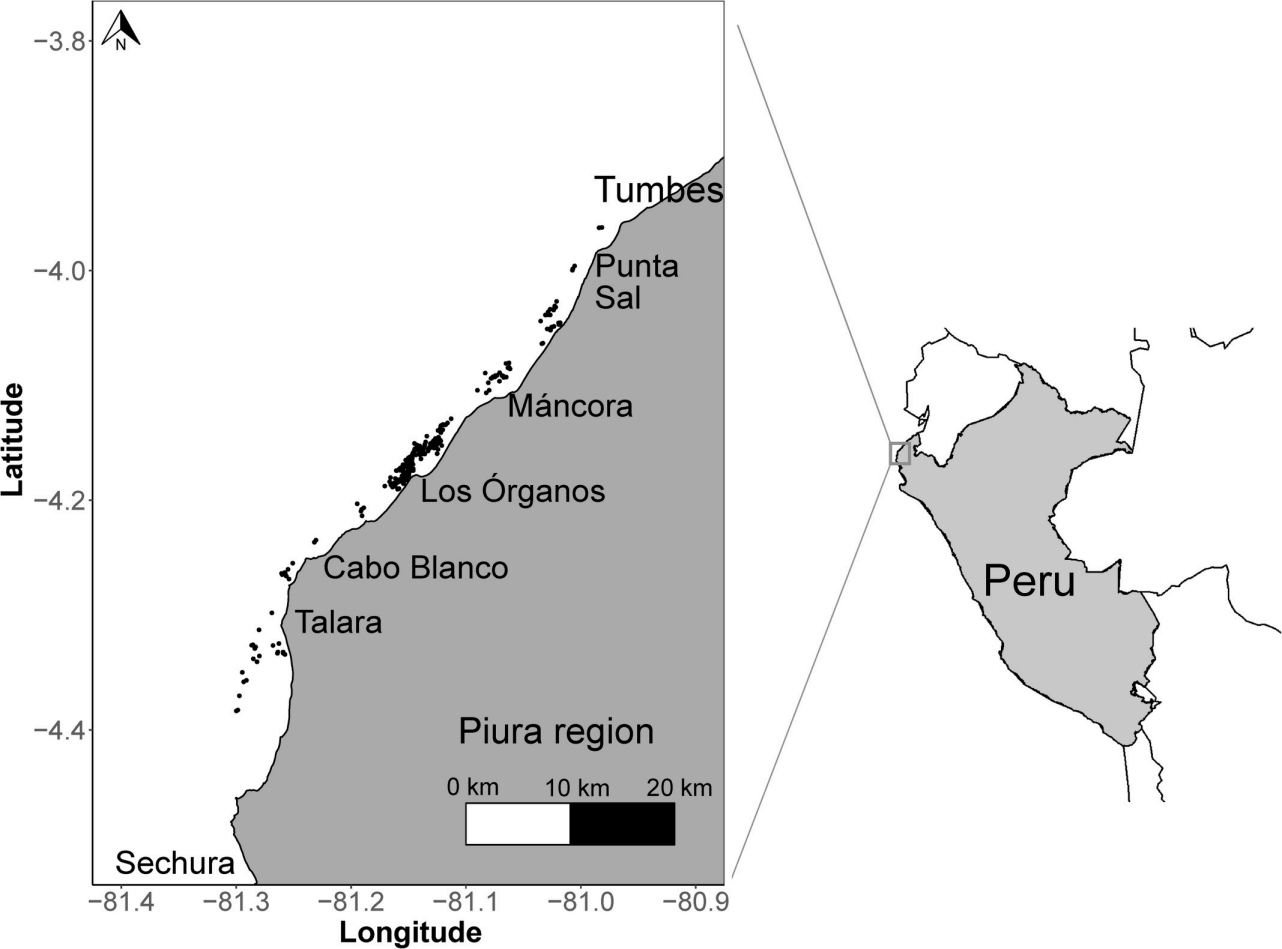
Location of fishing hauls carried out by the artisanal trawl fleet leaving port between Cabo Blanco and Máncora between April 2019—March 2020.

The study focused in the area between Cabo Blanco and Máncora (Fig 1). This fishery is prosecuted by small scale fishing vessels between 8.4 and 13.5m in length [14], operating within 5 miles of the coast in water depths of 6.5 to 65m [10]. The vessels have a hold capacity of 5 to 10 t, and 90% of them operate with a central engine whose fuel consumption is 10 to 20 gallons per day [15]. Around 30 vessels are equipped with a winch, echo sounder and satellite navigator [16]. Fishing occurs at night and mostly during the hours of darkness in calm weather conditions using polyamide nets 18 to 24 m total length with a codend mesh size of 32 to 40 mm. Haul durations are variable but generally last around two hours [10].

On-board observers collected data between April 2019 and March 2020 from 22 vessels. Data from 300 hauls were collected together with vessel track and gear deployment time and positions using a bespoke Android mobile telephone application (App) created for the project [17]. For each haul, the weight of the net and the catch including the net were recorded with a digital scale (KAMBOR 1 tonne capacity, +/- 0.5 kg accuracy). The total catch weight was calculated by subtracting the weight of the net from the weight of the total catch and net. The total catch consists of the target species coffee shrimp *Penaeus californiensis* and bycatch, defined by [5] as the sum of discarded organisms and non-target commercial species that are retained and marketed or consumed. In the case of this study commercial species included species that were sold and that were exchanged for other products or services. When the catch was on-board, the fishers placed the coffee shrimp and the commercial species into 0.08 m^3^ PVC boxes, which were weighed by the observers. The remaining catch constituted the discards and for each haul, a sample (on average 11.0 +/-2.4 kg SD) was taken using an 18l capacity bucket and weighed with a digital scale (KAMBOR 100 kg capacity, +/-20 gr accuracy). This sample represented on average 14.0% of the bycatch weight +/-8.9 kg SD. The number and weight of individuals by species or taxon were recorded and weighed and their total weight and number estimated as function of the total catch. Species were identified using: [18–25]. The FishBase (https://www.fishbase.se/search.php), World Register of Marine Species (WoRMS, http://www.marinespecies.org/) and SeaLifeBase (https://www.sealifebase.ca/) databases were also used for species identification.

Total discards were estimated as follows:

Total discards = Total catch–(coffee shrimp + commercial catch)

Using the capture data and the time spent trawling in hours, Capture per Unit Effort was estimated (CPUE, kg.h^-1^) for each component of the catch.

To assess changes in CPUE (kg.h^-1^) over time for each catch component (coffee shrimp, commercial catch, discards) an Analysis of Variance was conducted, with month specified as a factor. However, as we expected temporal autocorrelation in CPUE over time, we examined plots of autocorrelation functions [26]. Autocorrelation was evident for all components of the catch, therefore we used Generalised Least Squares models (GLS), to account for auto-correlation using the R package nlme [27]. We specified an order-1 autoregressive moving average (ARMA) correlation structure in the model, as we expected stronger temporal autocorrelation for months closer to each other. Differences in spread in the data for each month was addressed by using the varIdent structure [28]. Assumptions of normality of residuals and homogeneity of variances were assessed visually, and autocorrelation plots were examined again to confirm lack of significant autocorrelation in the model residuals. An Analysis of Variance was conducted on results from the Generalised Least Squares model to assess the overall effect of time. For all hypothesis testing, p values less than 0.05 were considered to represent significant evidence against the null hypotheses. Significant differences between months were assessed using the R package multcomp [29].

## Results

### Species composition

During the study period from April 2019 –March 2020, 277 species were recorded. 111 species of fish (40.1%); 65 species of molluscs (23.5%); 51 species of crustaceans (18.4%); 22 species of algae (7.9%); 12 species of cnidarian (4.3%), 9 species of echinoderms (3.2%), 4 species of Bryozoa (1.4%) and 3 species of polychaete (1.1%).

**Fig 2:**
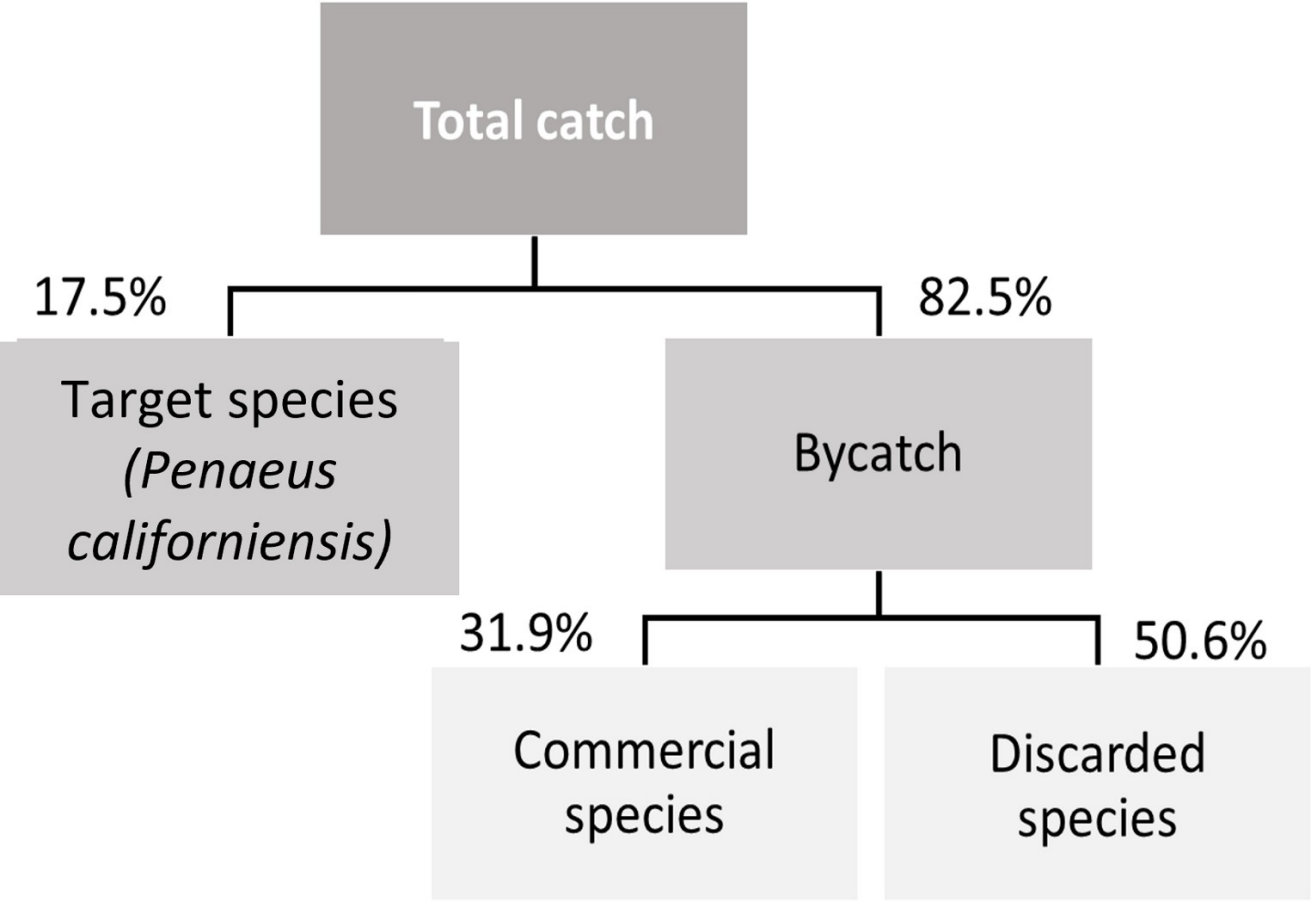
Catch composition showing the contributions of target species, bycatch, commercial species and discards to the total CPUE.

Total coffee shrimp catch constituted 17.8% of the total CPUE and bycatch constituted 82.2% (Fig 2). In the bycatch, non-target commercial species accounted on average for 30.1% of the total CPUE.

The target species coffee shrimp had the highest average CPUE with 14.8 kg.h^-1^; followed by sand perch *(Diplectrum conceptione)* with 13.5 kg.h^-1^; macroalgae caulerpa *(Caulerpa filiformis)* with 10.8 kg.h^-1^; sole flounder *(Etropus ectenes)* with 5.3 kg.h^-1^; Pacific drum (*Larimus pacificus*) with 4.7 kg.h^-1^ and the lumptail searobin (*Prionotus stephanophrys*) with 4.2 kg.h^-1^ (S1 Data). A total of 31 commercial species, including the target species, were retained for marketing, consumption or barter. Of these, the most abundant species were sand perch (*Diplectrum conception)*, 16.3% and sole flounder (*Etropus ectenes)*, 6.4% (S1 Data). The other commercial species constituted a small percentage of the total CPUE (S1 Data).

The discarded species accounted for 50.1% of the total CPUE (Fig 2). The main species were the macroalgae *caulerpa*, Pacific drum, lumptail searobin, swimming crab (*Portunus stephanophrys*), daisy midshipman (*Porichthys margaritatus*), and the crab *(Hepatus kossmanni*). A total of 246 species were discarded, of these, all species of macroalgae, cnidaria, echinoderms and polychaetes, 93.8% of molluscs, 88.2% of crustacean and 81.1% of fish species were discarded (S1 Data).

### Temporal variation in catches

**Fig 3:**
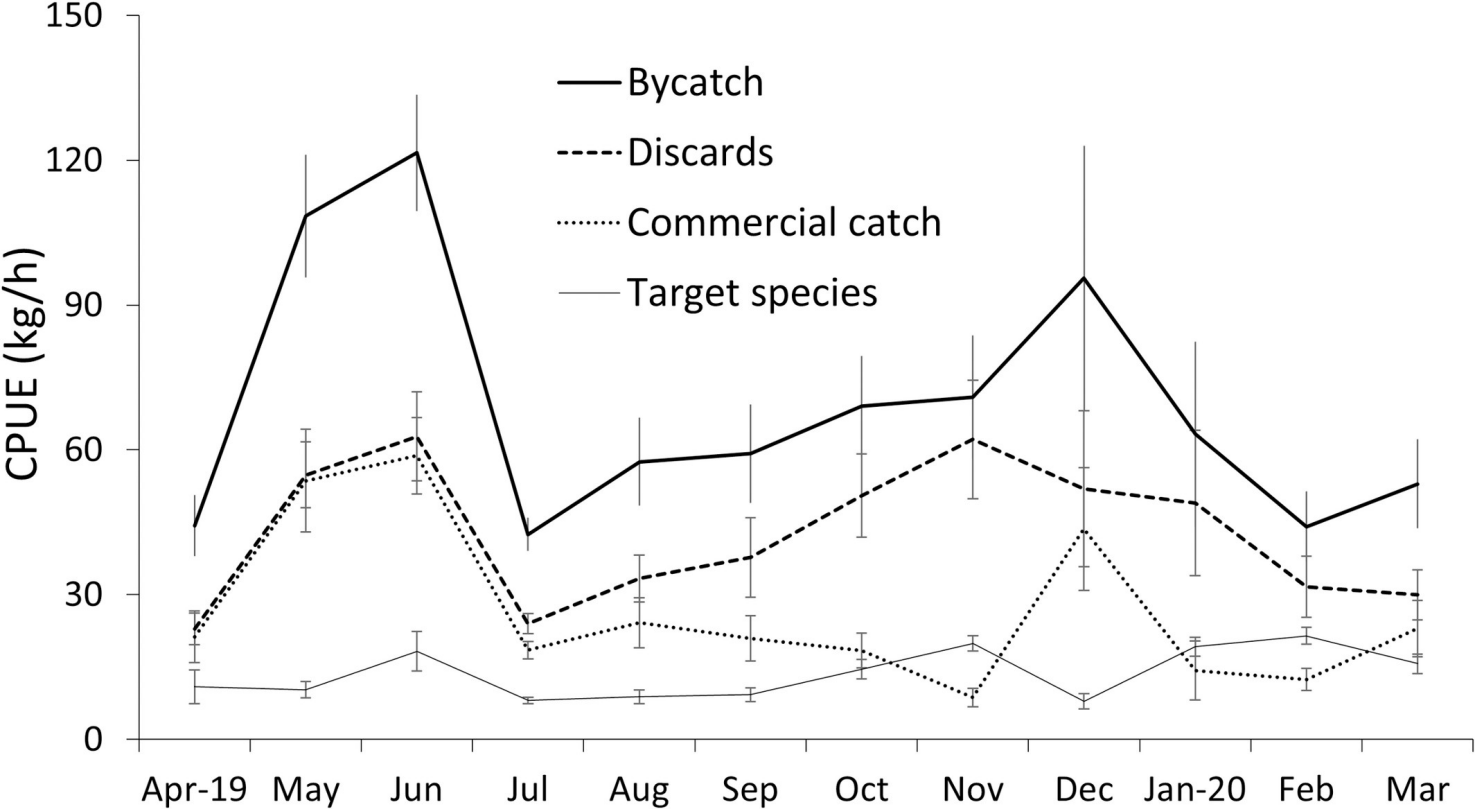
Monthly variation of the average CPUE (kg.h^-1^) of bycatch, discards, target species, and commercial species caught by the shrimp trawlers between Cabo Blanco and Máncora, April 2019-March 2020. Error bars represent standard errors.

The mean monthly CPUE of the coffee shrimp ranged between 7.9 and 21.5 kg.h^-1^, for commercial species CPUE between 8.7–58.8 kg.h^-1^, and for bycatch between 42.5 to 121.5 kg.h^-1^ (Fig 3). Significant differences in CPUE between months were found for coffee shrimp and commercial species catch (Table 1). No significant differences were detected between months for bycatch or discards CPUE (Table 1) due to the high variability in the data. Generalised Least Squares Model results and differences between months are presented in S1 File.

**Table 1:** Results from Generalised Least Squares model to assess overall effects of time on CPUE.

	Sample size	F-test		
	*n*	*df*	F	*p*-value
**Coffee shrimp CPUE**	300	11	5.4	<0.001
**Commercial species CPUE**	300	11	2.72	0.002
**Bycatch CPUE**	300	11	1.54	0.11
**Discards CPUE**	300	11	1.09	0.36

The months with the highest coffee shrimp CPUE were November 2019 and January/-February 2020, while lowest catches occurred in July, August and December (S1 File). Highest values of bycatch CPUE were recorded in May, June and December 2019 and lowest values in July 2019, February and March 2020 (S1 File). With the exception of November 2019 and January-February 2020, in all months the mean capture of non-target commercial species exceeded the capture of coffee shrimp (Fig 3).

### Bycatch to coffee shrimp ratio

**Fig 4:**
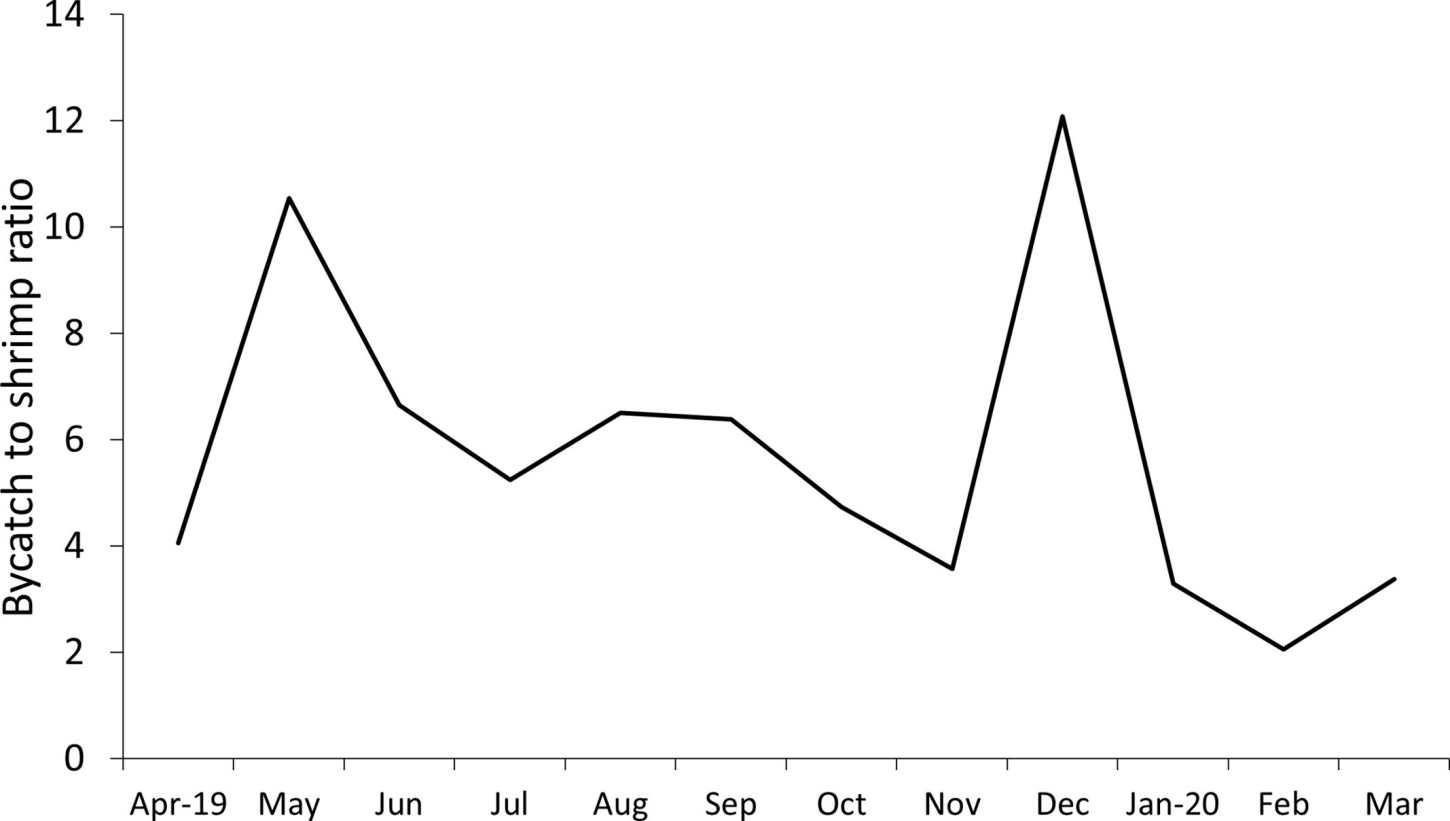
Ratio of aggregated bycatch and target species CPUE in the shrimp trawl fishery operating between Cabo Blanco and Máncora, April 2019-Mar 2020.

The bycatch to coffee shrimp ratio was on average 5.7:1, i.e. around 6 kg of bycatch are caught for each kg of coffee shrimp. The highest values of 10:5 and 12:1 in this study occurred in May and December 2019 respectively (Fig 4). In the remaining months, the average was 4.7:1, but with a noticeable reduction during the months of January-March 2020.This reduction in bycatch to coffee shrimp ratio during the summer months, is influenced by an increase in shrimp catches (Fig 4).

## Discussion

The number of species caught during this study is much higher than reported previously for Peruvian shrimp trawl fisheries. [12, 30] reported 10 and 83 species respectively), albeit their fieldwork lasted less than a month. Compared to previous studies of similar magnitude in tropical and subtropical waters, the number of species were similar, with for example 318 species recorded in the Gulf of California (Mexico) during 2004–2005 [31]; 261 in Ecuador’s shrimp trawling fleet [32]; 253 in the Colombian Caribbean during 2000–2001 [33] and 281 recorded on the southwest coast of India [34]. The average CPUE across the 300 hauls, shows fish as the most abundant in catches, followed by crustaceans, macroalgae and molluscs, which follows similar patterns observed in Tumbes, Peru [16, 30, 35] and in other countries [31, 33, 36, 37].

The percentage bycatch in this study (82%) mirrors similar research in other tropical and subtropical countries where coastal shrimp trawl fishery is permitted legally, such as [38] reporting 87.15% for the Colombian Caribbean, between 86–99% in Kuwait [39], 86% for Cuba’s South East Platform [37], 90% in Trinidad and Tobago [7], 86% in Norway [40], 84% in the Gulf of Mexico [41], 70% to 90% in India [34], and 70% to 80% in Ecuador [32]. In Peru, in Sechura Bay, Piura, 93.3% of the total catch was estimated as bycatch in a 15-day fishing survey in May 2010 [12]. In the Tumbes region bycatch between 44% and 79% was reported by [30] and 95% on average was reported by [11]. Although the bycatch of shrimp fisheries in Peru is similar to that in other countries, its illegality does not allow routine data collection. In this context, and in a scenario of weak control and surveillance, the impact of this fishery is probably greater than in other countries, where there is information and management measures can be proposed to reduce the impact.

Protected, endangered and threatened species have been reported as part of the bycatch in shrimp trawl fisheries in other regions of the world [5, 42]. No marine mammals were captured in this study, but three green turtles *(Chelonia mydas)* classified as endangered according to the IUCN red list [43] were caught in three different hauls, which were discarded alive. In addition, the seahorse *(Hippocampus ingens)*, a vulnerable species according to the IUCN red list [44] was recorded in 102 out of the 300 hauls, however, on average, only 1–2 individuals per haul were caught. Peru is known to represent the largest seahorse trade in South America and it is associated with the traditional Chinese medicine [45]. In 2019, the police seized around 12.34 million dried seahorses in the Peruvian port of Callao, from where they were going to be sent to Asia, with an estimated value of US$ 6 million [46]. Although the shrimp trawlers have been previously associated with the dry seahorse trade, it was beyond the scope of this study to assess whether the small-scale shrimp trawl fisheries contribute to this trade.

Although the FAO [5] defines discards or discarded catches as the part of the total organic material of animal origin in the catch, which is thrown into the sea for any reason. However, this does not include macroalgae which have been considered in this study as they are important in the structure and functioning of the ecosystem. Macroalgae were predominant in spring and summer (October to March), and accounted for about half of the discard CPUE. Discards in this study (50.6% of CPUE) were higher than the 19.1% reported by [30] and lower than the 95% reported by [11] for the Tumbes region. However, in the latter work, sand perch was not considered as a commercial species, but as a discard, because at that time this species was not consumed. According to [5], tropical shallow water shrimp fisheries like the one in Peru, account for 70% of the total estimated discards from shrimp trawl fisheries, represented mainly by penaeid shrimp fisheries. These fisheries have an average discard rate of 67.8%, which is higher than found in this study, however discard rates can vary considerably between countries. Fisheries in the Gulf of Mexico (56.9%), Venezuela (60%), Ecuador (79.1%), Indonesia (81.7%), and in Atlantic United States (83.3%), account for discard rates higher than reported in this study. A broader discussion on the implementation of a management plan for the fishery in Peru taking the experiences and knowledge in other countries of the region should be considered in order to reduce the impact on biodiversity.

Of the 246 species discarded, 46 were species that are marketed or consumed in other countries around the world. However, due to their sizes or lack of acceptance by the consumer in Peru they were not retained. Among these are the: Pacific drum; lumptail searobin; rough swimcrab; crab *Hepatus kossmanni*; squilla (*Squilla panamensis*); sole (*Symphurus* sp.); smooth stargazer (*Kathetostoma averruncus*); rock shrimp (*Sicyonia disdorsalis*) and Ferguson’s cone snail *(Conus fergusoni*). Further work needs to be conducted to see whether some of these species might potentially be used or consumed and how to change consumer acceptance. This seems to be the case already for sand perch, which only a decade ago was not widely consumed [11] but is now one of the main commercial species in the fishery.

The target species for the small-scale trawl fishery in northern Peru was originally considered to be coffee shrimp. However, this study confirms that, in addition to shrimp, other species such as the sand perch and the sole flounder have become important commercial species in this fishery. Overall, the contribution of these two non-target species exceeded the weight of the target species. The interpretation that shrimps are the sole target species therefore needs to be revised considering the volumes of small fish in the catch and their importance in consumption at the local and regional level. In this regard [30], considered lumptail searobin and sand perch as target species in shrimp trawling in the Tumbes region based on the high catch volumes of these species. Whilst shrimps represent the highest value per unit weight [10] sand perch and sole flounder, subject to volume and market price are clearly of sufficient value to fishers to be retained as part of the catch.

The bycatch to coffee shrimp ratio (5.7:1) was lower than in the trawler fleets of Kuwait which recorded an average ratio of 15:1 [47], Cuba 7:1 [37] and Nigeria from 8:1 to 15:1 [7]. In Ecuador, fish bycatch alone has 4.4:1 to 11.7:1 [32], however, using average data from 1998–2009, this ratio was 10:1 [48]. According to [33] bycatch: shrimp ratio values in the Colombian Caribbean ecosystem are among the highest in the world with 10.8:1 to 16.6:1 but similar values are reported for Venezuela and Mexico [31]. More recent estimates of shrimp trawler bycatch in the Gulf of Mexico show a relatively low ratio of 4.5:1 to 1:1 and 5.25:1 as a result of bycatch reduction measures being incorporated into trawl nets [7]. Similarly, low bycatch values of 1.4–6.4:1 have been reported in the Arabian Sea [39]. Bycatch:shrimp ratio shows strong variations which could be related to season (windy and calm) and depth as showed by [38] and with lunar cycle [49] which should be explored in future studies in the shrimp fishery in northern Peru.

Initiatives to reduce the bycatch in Latin American shrimp trawl fisheries by modifying fishing nets have been conducted recently, in a 5-year FAO project (Proyecto Gestión Sostenible de la Captura Incidental en la Pesca de Arrastre de América Latina y el Caribe, REBYC II LAC). Between 2015–2020 bycatch reduction trials were conducted in Mexico, Colombia, Brasil, Costa Rica, Suriname and Trinidad and Tobago. The levels of reduction in bycatch were between 16 and 46% (http://www.fao.org/in-action/rebyc-2/es/). Similar results have been obtained in the DYNAMICO-PERU project, in which valuable biological and behavioural information of non-target species was provided for the design of a modified trawl net that reduces bycatch rates by ~50% without affecting shrimp catches or economic returns [10].

The results of this study are the first reported for the Piura region and through analyses of the composition and abundance of species in the catch, it provides the basis for starting to assess the magnitude of the impact of the shrimp trawl fleet on the species community. As mentioned above, the fraction of the non-target and discarded species catch is in the range of similar fisheries in other countries where the use of this trawling method is permitted (e.g. Colombia, Ecuador, Mexico). However, the level of impact on stocks or species that could jeopardize their productivity in this ecosystem are unknown. In the 1980s and 1990s, overfishing of red snapper (*Lutjanus campechanus*), a commercially valuable species, was attributed to the bycatch of juvenile individuals in shrimp trawling in the Gulf of Mexico [50], however [51] concluded that the bycatch of red snapper would only reduce the annual allowable catch in other fisheries ~by 1% and have no impact on population growth. Future studies should aim to assess the impact of shrimp trawling on biodiversity considering the role of discarded species in the ecosystem, and their biological characteristics. These studies should cover aspects related to the life history of the captured species, the trophic relationships between them and the application of ecotrophic models such as ECOPATH and ECOSIM [52–55]. These approaches would facilitate the identification of measures to conserve the structure and functioning of the ecosystem and the goods and services it offers.

The growth and persistence of shrimp trawling for more than three decades in a scenario with weak control, albeit with increasingly harsh penalties for fishers subject to prosecution can be explained in economic terms. The relatively high income of the vessel owners and fishers operating in this fishery offers a strong incentive even when they are subject to the seizure of the nets, and to the threat of up to four years of imprisonment (D.L. N° 1393). Fishers have lobbied the Peruvian authorities to decriminalise the fishery within the 5 NM and have expressed willingness to comply with any required gear modifications for bycatch reduction. However, there is clearly a dichotomy between national legislation (General Fisheries Law 2001, DS-012-2001-PE) which part of civil society supports and is in favour of the closure of this fishery and ineffectual policing and compliance because the fishery is economically lucrative and provides a degree of local food security. James *et al*., (in prep) estimate the gross value of the fishery to be ~$10.6 m (US) annually with captains and deckhands, earning more than 3 times the “living wage” as defined in the 2017 census and of the order of 50% higher than the average monthly income quoted for men in Lima [56]. In a regulatory regime where there are great deficiencies in control and surveillance, lack of decision-making supports the status-quo, namely the persistence of this fishery inside 5NM, with high levels of bycatch.

Given that the fishery remains illegal, implementation of management and conservation measures using a top-down approach (e.g. from government) is unlikely. In the absence of effective policing, the fishery will continue and therefore pragmatism should perhaps dictate that those with greatest interest in protecting the fishery are the fishers themselves. As fishers are interested in reducing their impact on the ecosystem to try to initiate dialogue with government, a combination of temporary closures and the use of modified nets could be self-regulated by fishers. For example, the months of May and December had the greatest bycatch to shrimp ratio and the fishery could potentially be closed to avoid high bycatch risk, however, longer term information is needed to assess if these trends persist over time. Management measures should be identified and discussed in a co-management participatory process to achieve the best outcomes for the sustainability of the target species and their ecosystem. Encouragingly, in July 2021, fishers asked in members of the DYNAMICOPERU project for a training workshop to learn how to construct the modified nets themselves. This took place in March 2022, with encouraging results.

## Supporting information

S1 File (DOCX)

S1 Data (XLSX)
