# Supplementary material for: Bycatch and discards in the artisanal shrimp trawl fishery in Northern Peru
Source: PLoS One. 2022 Jun 22;17(6):e0268128. doi: 10.1371/journal.pone.0268128 (PMC9216530; doi:10.1371/journal.pone.0268128)
Supplement: S1 File — (DOCX) [file pone.0268128.s001.docx]

***Temporal variation in catches of coffee shrimp***

Table S1. Coefficients for the final generalised least squares models with corresponding standard errors, t and p-values for variation in coffee shrimp CPUE over time (months).

| Model term | Value | St. Error | t-value | p-value |
| --- | --- | --- | --- | --- |
| Intercept | 19.690 | 2.390 | 8.236 | **0.000** |
| February | 0.775 | 3.095 | 0.250 | 0.802 |
| March | -3.389 | 3.713 | -0.912 | 0.362 |
| April | -7.800 | 6.053 | -1.288 | 0.198 |
| May | -9.068 | 3.431 | -2.643 | **0.008** |
| June | -1.946 | 6.021 | -0.323 | 0.746 |
| July | -11.639 | 2.627 | -4.429 | **0.000** |
| August | -10.886 | 3.077 | -3.538 | **0.000** |
| September | -9.425 | 3.082 | -3.058 | **0.002** |
| October | -5.139 | 3.549 | -1.447 | 0.148 |
| November | -0.242 | 3.395 | -0.071 | 0.943 |
| December | -11.307 | 3.131 | -3.611 | **0.000** |

Table S2. Tukey contrasts, with corresponding estimate coefficients, standard errors, Z and p-values for comparisons between means in coffee shrimp CPUE between months

| Month comparison | Estimate | St. error | z-value | p-value |
| --- | --- | --- | --- | --- |
| 2-1 | 0.775 | 3.095 | 0.250 | 1.000 |
| 3-1 | -3.389 | 3.714 | -0.913 | 0.999 |
| 4-1 | -7.800 | 6.054 | -1.289 | 0.976 |
| 5-1 | -9.069 | 3.431 | -2.643 | 0.230 |
| 6-1 | -1.947 | 6.021 | -0.323 | 1.000 |
| 7-1 | -11.639 | 2.628 | -4.429 | **0.001** |
| 8-1 | -10.887 | 3.077 | -3.538 | **0.017** |
| 9-1 | -9.425 | 3.082 | -3.058 | 0.081 |
| 10-1 | -5.139 | 3.550 | -1.448 | 0.945 |
| 11-1 | -0.243 | 3.396 | -0.072 | 1.000 |
| 12-1 | -11.308 | 3.131 | -3.611 | **0.014** |
| 3-2 | -4.164 | 3.456 | -1.205 | 0.986 |
| 4-2 | -8.575 | 6.020 | -1.425 | 0.951 |
| 5-2 | -9.844 | 3.371 | -2.920 | 0.118 |
| 6-2 | -2.722 | 5.987 | -0.455 | 1.000 |
| 7-2 | -12.414 | 2.549 | -4.871 | **0.000** |
| 8-2 | -11.662 | 3.010 | -3.875 | **0.005** |
| 9-2 | -10.200 | 3.015 | -3.383 | **0.029** |
| 10-2 | -5.915 | 3.492 | -1.694 | 0.853 |
| 11-2 | -1.018 | 3.339 | -0.305 | 1.000 |
| 12-2 | -12.083 | 3.180 | -3.799 | **0.007** |
| 4-3 | -4.411 | 6.264 | -0.704 | 1.000 |
| 5-3 | -5.679 | 3.790 | -1.499 | 0.931 |
| 6-3 | 1.443 | 6.232 | 0.231 | 1.000 |
| 7-3 | -8.250 | 3.081 | -2.677 | 0.213 |
| 8-3 | -7.497 | 3.472 | -2.159 | 0.545 |
| 9-3 | -6.036 | 3.477 | -1.736 | 0.830 |
| 10-3 | -1.750 | 3.898 | -0.449 | 1.000 |
| 11-3 | 3.146 | 3.763 | 0.836 | 0.999 |
| 12-3 | -7.918 | 3.679 | -2.152 | 0.550 |
| 5-4 | -1.268 | 5.924 | -0.214 | 1.000 |
| 6-4 | 5.854 | 7.830 | 0.748 | 1.000 |
| 7-4 | -3.839 | 5.666 | -0.678 | 1.000 |
| 8-4 | -3.086 | 5.784 | -0.534 | 1.000 |
| 9-4 | -1.625 | 5.880 | -0.276 | 1.000 |
| 10-4 | 2.661 | 6.148 | 0.433 | 1.000 |
| 11-4 | 7.557 | 6.066 | 1.246 | 0.982 |
| 12-4 | -3.507 | 6.020 | -0.583 | 1.000 |
| 6-5 | 7.122 | 5.963 | 1.194 | 0.987 |
| 7-5 | -2.571 | 2.691 | -0.955 | 0.998 |
| 8-5 | -1.818 | 3.126 | -0.582 | 1.000 |
| 9-5 | -0.357 | 3.137 | -0.114 | 1.000 |
| 10-5 | 3.929 | 3.598 | 1.092 | 0.994 |
| 11-5 | 8.826 | 3.453 | 2.556 | 0.277 |
| 12-5 | -2.239 | 3.372 | -0.664 | 1.000 |
| 7-6 | -9.693 | 5.604 | -1.730 | 0.833 |
| 8-6 | -8.940 | 5.854 | -1.527 | 0.922 |
| 9-6 | -7.479 | 5.858 | -1.277 | 0.978 |
| 10-6 | -3.193 | 6.118 | -0.522 | 1.000 |
| 11-6 | 1.704 | 6.033 | 0.282 | 1.000 |
| 12-6 | -9.361 | 5.987 | -1.563 | 0.909 |
| 8-7 | 0.753 | 2.195 | 0.343 | 1.000 |
| 9-7 | 2.214 | 2.227 | 0.994 | 0.997 |
| 10-7 | 6.500 | 2.842 | 2.287 | 0.451 |
| 11-7 | 11.396 | 2.656 | 4.291 | **0.001** |
| 12-7 | 0.332 | 2.550 | 0.130 | 1.000 |
| 9-8 | 1.461 | 2.589 | 0.564 | 1.000 |
| 10-8 | 5.747 | 3.242 | 1.773 | 0.809 |
| 11-8 | 10.644 | 3.100 | 3.433 | **0.025** |
| 12-8 | -0.421 | 3.010 | -0.140 | 1.000 |
| 10-9 | 4.286 | 3.080 | 1.392 | 0.959 |
| 11-9 | 9.182 | 3.101 | 2.961 | 0.106 |
| 12-9 | -1.882 | 3.015 | -0.624 | 1.000 |
| 11-10 | 4.896 | 3.518 | 1.392 | 0.958 |
| 12-10 | -6.168 | 3.491 | -1.767 | 0.813 |
| 12-11 | -11.065 | 3.283 | -3.370 | **0.031** |

***Temporal variation in bycatch***

Table S3. Coefficients for the final generalised least squares models with corresponding standard errors, t and p-values for variation in bycatch CPUE over time (months)

| Model term | Value | St. Error | t-value | p-value |
| --- | --- | --- | --- | --- |
| Intercept | 92.171 | 32.202 | 2.862 | 0.004 |
| February | -50.120 | 30.290 | -1.654 | 0.099 |
| March | -35.094 | 34.132 | -1.028 | 0.304 |
| April | -42.606 | 34.087 | -1.249 | 0.212 |
| May | 2.960 | 40.252 | 0.073 | 0.941 |
| June | 15.396 | 40.543 | 0.379 | 0.704 |
| July | -42.196 | 33.329 | -1.266 | 0.206 |
| August | -36.469 | 34.621 | -1.053 | 0.293 |
| September | -42.348 | 36.378 | -1.164 | 0.245 |
| October | -7.670 | 38.202 | -0.200 | 0.841 |
| November | -41.582 | 42.378 | -0.981 | 0.327 |
| December | 16.834 | 41.478 | 0.405 | 0.685 |

Table S4. Tukey contrasts, with corresponding estimate coefficients, standard errors, Z and p-values for comparisons between means in bycatch CPUE between months

| Month comparison | Estimate | St. error | z-value | p-value |
| --- | --- | --- | --- | --- |
| 2-1 | -50.121 | 30.291 | -1.655 | 0.863 |
| 3-1 | -35.094 | 34.133 | -1.028 | 0.996 |
| 4-1 | -42.606 | 34.087 | -1.250 | 0.980 |
| 5-1 | 2.960 | 40.253 | 0.074 | 1.000 |
| 6-1 | 15.397 | 40.544 | 0.380 | 1.000 |
| 7-1 | -42.197 | 33.330 | -1.266 | 0.977 |
| 8-1 | -36.469 | 34.621 | -1.053 | 0.995 |
| 9-1 | -42.348 | 36.378 | -1.164 | 0.988 |
| 10-1 | -7.671 | 38.202 | -0.201 | 1.000 |
| 11-1 | -41.583 | 42.379 | -0.981 | 0.997 |
| 12-1 | 16.835 | 41.478 | 0.406 | 1.000 |
| 3-2 | 15.026 | 15.633 | 0.961 | 0.998 |
| 4-2 | 7.514 | 15.428 | 0.487 | 1.000 |
| 5-2 | 53.081 | 26.388 | 2.012 | 0.641 |
| 6-2 | 65.517 | 26.830 | 2.442 | 0.333 |
| 7-2 | 7.924 | 13.672 | 0.580 | 1.000 |
| 8-2 | 13.652 | 16.579 | 0.823 | 0.999 |
| 9-2 | 7.773 | 20.006 | 0.389 | 1.000 |
| 10-2 | 42.450 | 23.209 | 1.829 | 0.766 |
| 11-2 | 8.538 | 30.280 | 0.282 | 1.000 |
| 12-2 | 66.956 | 36.423 | 1.838 | 0.759 |
| 4-3 | -7.512 | 19.261 | -0.390 | 1.000 |
| 5-3 | 38.054 | 28.798 | 1.321 | 0.969 |
| 6-3 | 50.491 | 29.203 | 1.729 | 0.825 |
| 7-3 | -7.102 | 17.886 | -0.397 | 1.000 |
| 8-3 | -1.375 | 20.196 | -0.068 | 1.000 |
| 9-3 | -7.254 | 23.094 | -0.314 | 1.000 |
| 10-3 | 27.423 | 25.923 | 1.058 | 0.995 |
| 11-3 | -6.488 | 32.489 | -0.200 | 1.000 |
| 12-3 | 51.929 | 39.073 | 1.329 | 0.968 |
| 5-4 | 45.567 | 24.496 | 1.860 | 0.745 |
| 6-4 | 58.003 | 26.774 | 2.166 | 0.525 |
| 7-4 | 0.410 | 13.982 | 0.029 | 1.000 |
| 8-4 | 6.137 | 15.564 | 0.394 | 1.000 |
| 9-4 | 0.258 | 19.878 | 0.013 | 1.000 |
| 10-4 | 34.936 | 23.342 | 1.497 | 0.926 |
| 11-4 | 1.024 | 30.650 | 0.033 | 1.000 |
| 12-4 | 59.441 | 38.399 | 1.548 | 0.908 |
| 6-5 | 12.437 | 32.203 | 0.386 | 1.000 |
| 7-5 | -45.157 | 25.512 | -1.770 | 0.801 |
| 8-5 | -39.429 | 26.929 | -1.464 | 0.936 |
| 9-5 | -45.308 | 29.376 | -1.542 | 0.910 |
| 10-5 | -10.631 | 31.729 | -0.335 | 1.000 |
| 11-5 | -44.543 | 37.393 | -1.191 | 0.986 |
| 12-5 | 13.875 | 43.965 | 0.316 | 1.000 |
| 7-6 | -57.593 | 25.307 | -2.276 | 0.446 |
| 8-6 | -51.866 | 27.577 | -1.881 | 0.732 |
| 9-6 | -57.745 | 29.850 | -1.934 | 0.695 |
| 10-6 | -23.068 | 32.125 | -0.718 | 1.000 |
| 11-6 | -56.979 | 37.709 | -1.511 | 0.921 |
| 12-6 | 1.438 | 44.232 | 0.033 | 1.000 |
| 8-7 | 5.728 | 14.653 | 0.391 | 1.000 |
| 9-7 | -0.151 | 18.792 | -0.008 | 1.000 |
| 10-7 | 34.526 | 22.298 | 1.548 | 0.908 |
| 11-7 | 0.614 | 29.812 | 0.021 | 1.000 |
| 12-7 | 59.032 | 37.729 | 1.565 | 0.902 |
| 9-8 | -5.879 | 18.003 | -0.327 | 1.000 |
| 10-8 | 28.798 | 23.260 | 1.238 | 0.981 |
| 11-8 | -5.114 | 31.158 | -0.164 | 1.000 |
| 12-8 | 53.304 | 38.867 | 1.371 | 0.959 |
| 10-9 | 34.677 | 22.291 | 1.556 | 0.906 |
| 11-9 | 0.765 | 32.764 | 0.023 | 1.000 |
| 12-9 | 59.183 | 40.410 | 1.465 | 0.936 |
| 11-10 | -33.912 | 33.568 | -1.010 | 0.996 |
| 12-10 | 24.506 | 41.952 | 0.584 | 1.000 |
| 12-11 | 58.418 | 44.008 | 1.327 | 0.968 |

***Temporal variation in commercial catches***

Table S5. Coefficients for the final generalised least squares models with corresponding standard errors, t and p-values for variation in commercial catches CPUE over time (months).

| Model term | Value | St. Error | t-value | p-value |
| --- | --- | --- | --- | --- |
| Intercept | 19.307 | 8.499 | 2.271 | 0.023 |
| February | -7.640 | 8.699 | -0.878 | 0.380 |
| March | 4.594 | 11.555 | 0.397 | 0.691 |
| April | 1.332 | 10.611 | 0.125 | 0.900 |
| May | 31.546 | 18.362 | 1.718 | 0.086 |
| June | 36.049 | 15.025 | 2.399 | **0.017** |
| July | 0.218 | 9.130 | 0.023 | 0.980 |
| August | 3.590 | 10.610 | 0.338 | 0.735 |
| September | 1.828 | 10.327 | 0.177 | 0.859 |
| October | -0.249 | 9.850 | -0.025 | 0.979 |
| November | -11.013 | 9.062 | -1.215 | 0.225 |
| December | 28.730 | 17.755 | 1.618 | 0.106 |

Table S6. Tukey contrasts, with corresponding estimate coefficients, standard errors, Z and p-values for comparisons between means in commercial catches CPUE between months

| Month comparison | Estimate | St. error | z-value | p-value |
| --- | --- | --- | --- | --- |
| 2-1 | -7.641 | 8.700 | -0.878 | 0.999 |
| 3-1 | 4.595 | 11.556 | 0.398 | 1.000 |
| 4-1 | 1.332 | 10.612 | 0.126 | 1.000 |
| 5-1 | 31.546 | 18.362 | 1.718 | 0.829 |
| 6-1 | 36.050 | 15.026 | 2.399 | 0.356 |
| 7-1 | 0.219 | 9.130 | 0.024 | 1.000 |
| 8-1 | 3.591 | 10.611 | 0.338 | 1.000 |
| 9-1 | 1.828 | 10.328 | 0.177 | 1.000 |
| 10-1 | -0.249 | 9.851 | -0.025 | 1.000 |
| 11-1 | -11.013 | 9.062 | -1.215 | 0.984 |
| 12-1 | 28.731 | 17.756 | 1.618 | 0.878 |
| 3-2 | 12.235 | 8.319 | 1.471 | 0.933 |
| 4-2 | 8.973 | 7.371 | 1.217 | 0.983 |
| 5-2 | 39.187 | 16.700 | 2.347 | 0.391 |
| 6-2 | 43.690 | 12.942 | 3.376 | **0.028** |
| 7-2 | 7.859 | 5.008 | 1.569 | 0.899 |
| 8-2 | 11.232 | 7.369 | 1.524 | 0.916 |
| 9-2 | 9.469 | 6.956 | 1.361 | 0.961 |
| 10-2 | 7.391 | 6.227 | 1.187 | 0.986 |
| 11-2 | -3.373 | 4.911 | -0.687 | 1.000 |
| 12-2 | 36.371 | 16.997 | 2.140 | 0.542 |
| 4-3 | -3.262 | 10.262 | -0.318 | 1.000 |
| 5-3 | 26.952 | 18.163 | 1.484 | 0.930 |
| 6-3 | 31.455 | 14.781 | 2.128 | 0.549 |
| 7-3 | -4.376 | 8.722 | -0.502 | 1.000 |
| 8-3 | -1.004 | 10.261 | -0.098 | 1.000 |
| 9-3 | -2.766 | 9.969 | -0.278 | 1.000 |
| 10-3 | -4.844 | 9.474 | -0.511 | 1.000 |
| 11-3 | -15.608 | 8.669 | -1.800 | 0.781 |
| 12-3 | 24.136 | 18.579 | 1.299 | 0.973 |
| 5-4 | 30.214 | 16.932 | 1.784 | 0.791 |
| 6-4 | 34.718 | 13.898 | 2.498 | 0.295 |
| 7-4 | -1.113 | 7.166 | -0.155 | 1.000 |
| 8-4 | 2.259 | 8.650 | 0.261 | 1.000 |
| 9-4 | 0.496 | 8.603 | 0.058 | 1.000 |
| 10-4 | -1.582 | 8.067 | -0.196 | 1.000 |
| 11-4 | -12.345 | 7.113 | -1.736 | 0.819 |
| 12-4 | 27.399 | 17.984 | 1.524 | 0.916 |
| 6-5 | 4.503 | 19.948 | 0.226 | 1.000 |
| 7-5 | -31.328 | 16.608 | -1.886 | 0.725 |
| 8-5 | -27.955 | 17.433 | -1.604 | 0.885 |
| 9-5 | -29.718 | 17.296 | -1.718 | 0.829 |
| 10-5 | -31.796 | 17.021 | -1.868 | 0.737 |
| 11-5 | -42.560 | 16.588 | -2.566 | 0.256 |
| 12-5 | -2.816 | 23.409 | -0.120 | 1.000 |
| 7-6 | -35.831 | 12.716 | -2.818 | 0.142 |
| 8-6 | -32.459 | 13.914 | -2.333 | 0.402 |
| 9-6 | -34.222 | 13.708 | -2.496 | 0.295 |
| 10-6 | -36.299 | 13.354 | -2.718 | 0.182 |
| 11-6 | -47.063 | 12.796 | -3.678 | **0.010** |
| 12-6 | -7.319 | 20.894 | -0.350 | 1.000 |
| 8-7 | 3.372 | 7.056 | 0.478 | 1.000 |
| 9-7 | 1.610 | 6.732 | 0.239 | 1.000 |
| 10-7 | -0.468 | 5.992 | -0.078 | 1.000 |
| 11-7 | -11.232 | 4.620 | -2.431 | 0.336 |
| 12-7 | 28.512 | 17.151 | 1.662 | 0.857 |
| 9-8 | -1.763 | 8.016 | -0.220 | 1.000 |
| 10-8 | -3.840 | 7.989 | -0.481 | 1.000 |
| 11-8 | -14.604 | 7.109 | -2.054 | 0.604 |
| 12-8 | 25.140 | 17.983 | 1.398 | 0.953 |
| 10-9 | -2.078 | 7.110 | -0.292 | 1.000 |
| 11-9 | -12.841 | 6.666 | -1.926 | 0.697 |
| 12-9 | 26.902 | 17.816 | 1.510 | 0.921 |
| 11-10 | -10.764 | 5.814 | -1.851 | 0.748 |
| 12-10 | 28.980 | 17.537 | 1.652 | 0.862 |
| 12-11 | 39.744 | 16.979 | 2.341 | 0.396 |

***Temporal variation in discards***

Table S7. Coefficients for the final generalised least squares models with corresponding standard errors, t and p-values for variation in discards CPUE over time (months).

| Model term | Value | St. Error | t-value | p-value |
| --- | --- | --- | --- | --- |
| Intercept | 67.291 | 26.302 | 2.558 | 0.011 |
| February | -38.477 | 24.963 | -1.541 | 0.124 |
| March | -33.731 | 26.897 | -1.254 | 0.210 |
| April | -37.733 | 27.241 | -1.385 | 0.167 |
| May | -15.223 | 29.067 | -0.523 | 0.600 |
| June | -16.662 | 32.328 | -0.515 | 0.606 |
| July | -39.368 | 26.693 | -1.474 | 0.141 |
| August | -33.594 | 27.161 | -1.236 | 0.217 |
| September | -42.558 | 30.023 | -1.417 | 0.157 |
| October | -5.894 | 31.615 | -0.186 | 0.852 |
| November | -24.800 | 36.992 | -0.670 | 0.503 |
| December | -13.234 | 28.642 | -0.462 | 0.644 |

Table S8. Tukey contrasts, with corresponding estimate coefficients, standard errors, Z and p-values for comparisons between means in discards CPUE between months

| Month comparison | Estimate | St. error | z-value | p-value |
| --- | --- | --- | --- | --- |
| 2-1 | -38.478 | 24.963 | -1.541 | 0.907 |
| 3-1 | -33.732 | 26.897 | -1.254 | 0.978 |
| 4-1 | -37.734 | 27.241 | -1.385 | 0.954 |
| 5-1 | -15.223 | 29.067 | -0.524 | 1.000 |
| 6-1 | -16.662 | 32.329 | -0.515 | 1.000 |
| 7-1 | -39.369 | 26.694 | -1.475 | 0.930 |
| 8-1 | -33.595 | 27.161 | -1.237 | 0.980 |
| 9-1 | -42.559 | 30.024 | -1.418 | 0.946 |
| 10-1 | -5.895 | 31.615 | -0.186 | 1.000 |
| 11-1 | -24.800 | 36.992 | -0.670 | 1.000 |
| 12-1 | -13.234 | 28.642 | -0.462 | 1.000 |
| 3-2 | 4.746 | 10.555 | 0.450 | 1.000 |
| 4-2 | 0.744 | 11.619 | 0.064 | 1.000 |
| 5-2 | 23.255 | 15.422 | 1.508 | 0.919 |
| 6-2 | 21.816 | 20.930 | 1.042 | 0.995 |
| 7-2 | -0.891 | 10.270 | -0.087 | 1.000 |
| 8-2 | 4.883 | 11.432 | 0.427 | 1.000 |
| 9-2 | -4.081 | 17.168 | -0.238 | 1.000 |
| 10-2 | 32.583 | 19.853 | 1.641 | 0.864 |
| 11-2 | 13.678 | 28.115 | 0.486 | 1.000 |
| 12-2 | 25.244 | 20.842 | 1.211 | 0.983 |
| 4-3 | -4.002 | 11.368 | -0.352 | 1.000 |
| 5-3 | 18.509 | 15.233 | 1.215 | 0.983 |
| 6-3 | 17.070 | 20.791 | 0.821 | 0.999 |
| 7-3 | -5.637 | 9.984 | -0.565 | 1.000 |
| 8-3 | 0.137 | 11.177 | 0.012 | 1.000 |
| 9-3 | -8.827 | 17.001 | -0.519 | 1.000 |
| 10-3 | 27.837 | 19.714 | 1.412 | 0.948 |
| 11-3 | 8.932 | 28.107 | 0.318 | 1.000 |
| 12-3 | 20.498 | 21.661 | 0.946 | 0.998 |
| 5-4 | 22.510 | 13.071 | 1.722 | 0.822 |
| 6-4 | 21.071 | 19.938 | 1.057 | 0.995 |
| 7-4 | -1.635 | 8.369 | -0.195 | 1.000 |
| 8-4 | 4.139 | 9.069 | 0.456 | 1.000 |
| 9-4 | -4.825 | 15.883 | -0.304 | 1.000 |
| 10-4 | 31.839 | 18.901 | 1.684 | 0.843 |
| 11-4 | 12.933 | 27.628 | 0.468 | 1.000 |
| 12-4 | 24.499 | 21.414 | 1.144 | 0.989 |
| 6-5 | -1.439 | 21.262 | -0.068 | 1.000 |
| 7-5 | -24.146 | 13.131 | -1.839 | 0.752 |
| 8-5 | -18.372 | 13.942 | -1.318 | 0.968 |
| 9-5 | -27.335 | 18.986 | -1.440 | 0.941 |
| 10-5 | 9.328 | 21.493 | 0.434 | 1.000 |
| 11-5 | -9.577 | 29.434 | -0.325 | 1.000 |
| 12-5 | 1.989 | 23.694 | 0.084 | 1.000 |
| 7-6 | -22.707 | 18.957 | -1.198 | 0.985 |
| 8-6 | -16.932 | 19.914 | -0.850 | 0.999 |
| 9-6 | -25.896 | 23.703 | -1.093 | 0.993 |
| 10-6 | 10.767 | 25.741 | 0.418 | 1.000 |
| 11-6 | -8.138 | 32.660 | -0.249 | 1.000 |
| 12-6 | 3.428 | 27.598 | 0.124 | 1.000 |
| 8-7 | 5.774 | 7.828 | 0.738 | 1.000 |
| 9-7 | -3.190 | 15.089 | -0.211 | 1.000 |
| 10-7 | 33.474 | 18.151 | 1.844 | 0.749 |
| 11-7 | 14.569 | 27.092 | 0.538 | 1.000 |
| 12-7 | 26.135 | 20.713 | 1.262 | 0.977 |
| 9-8 | -8.964 | 14.242 | -0.629 | 1.000 |
| 10-8 | 27.700 | 18.361 | 1.509 | 0.919 |
| 11-8 | 8.794 | 27.511 | 0.320 | 1.000 |
| 12-8 | 20.361 | 21.310 | 0.955 | 0.998 |
| 10-9 | 36.664 | 19.302 | 1.899 | 0.711 |
| 11-9 | 17.758 | 30.062 | 0.591 | 1.000 |
| 12-9 | 29.324 | 24.838 | 1.181 | 0.986 |
| 11-10 | -18.906 | 30.660 | -0.617 | 1.000 |
| 12-10 | -7.339 | 26.681 | -0.275 | 1.000 |
| 12-11 | 11.566 | 31.847 | 0.363 | 1.000 |
